# Crystal structure of 1,1′-bis[1,7-dicarba-*closo*-dodeca­borane(11)]

**DOI:** 10.1107/S1600536814022132

**Published:** 2014-10-24

**Authors:** Lisa Elrick, Georgina M. Rosair, Alan J. Welch

**Affiliations:** a Institute of Chemical Sciences, School of Engineering & Physical Sciences, Heriot-Watt University, Edinburgh EH14 4AS, Scotland

**Keywords:** crystal structure, carboranes, *closo*-dodeca­borane(11)

## Abstract

In the title compound, C_4_H_22_B_20_, the two {1,7-*closo*-C_2_B_10_H_11_} cages are linked across a centre of inversion, with C—C = 1.5401 (16) Å. The position of the second non-linking cage C atom was established unambiguously by geometric and crystallographic methods and there is no evidence of C/B disorder.

## Abstract 2

In 1,1′-bis[1,7-dicarba-*closo*-dodeca­borane(11)], the two {1,7-*closo*-C_2_B_10_H_11_} cages are linked across a centre of inversion. The position of the second non-linking cage C atom was established unambiguously by geometric and crystallographic methods and there is no evidence of C/B disorder.

## Chemical context   

Whilst the chemistry of single-cage carboranes is well developed (Grimes, 2011 ▶) that of bis­(carboranes) (two discrete carborane units connected *via* a two-centre two-electron bond) is not. There are several isomeric possibilities for bis­(carboranes) composed of two C_2_B_10_ icosa­hedra. Bis(*ortho*-carborane), 1,1′-bis­[1,2-dicarba-*closo*-dodeca­borane(11)] (Dupont & Hawthorne, 1964 ▶), is the best known and its chemistry has been modestly developed (Hawthorne & Owen, 1971 ▶; Harwell *et al.*, 1996 ▶, 1997 ▶; Yanovsky *et al.*, 1979 ▶; Herzog *et al.*, 1999 ▶; Ellis *et al.*, 2010*a* ▶,*b* ▶). Bis(*meta*-carborane), 1,1′-bis[1,7-dicarba-*closo*-dodeca­borane(11)], the subject of this study is, however, less well known. It was first prepared by Zakharkin & Kovredov (1973 ▶) and later by Yang *et al.* (1995 ▶) with the latter authors providing ^1^H, ^13^C and ^11^B NMR spectroscopic and mass spectrometric analysis. We now report the structural study of the title compound, 1,1′-bis[1,7-dicarba-*closo*-dodeca­borane(11)], (I).
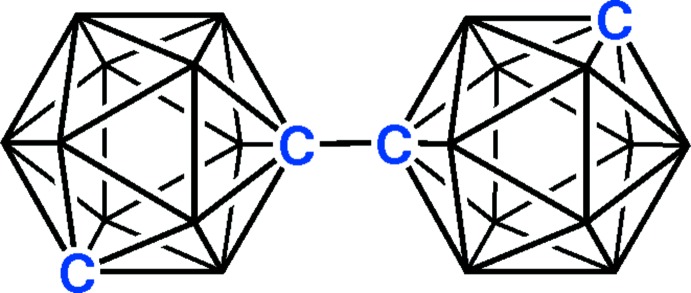


## Structural commentary   

**Figure 1:**
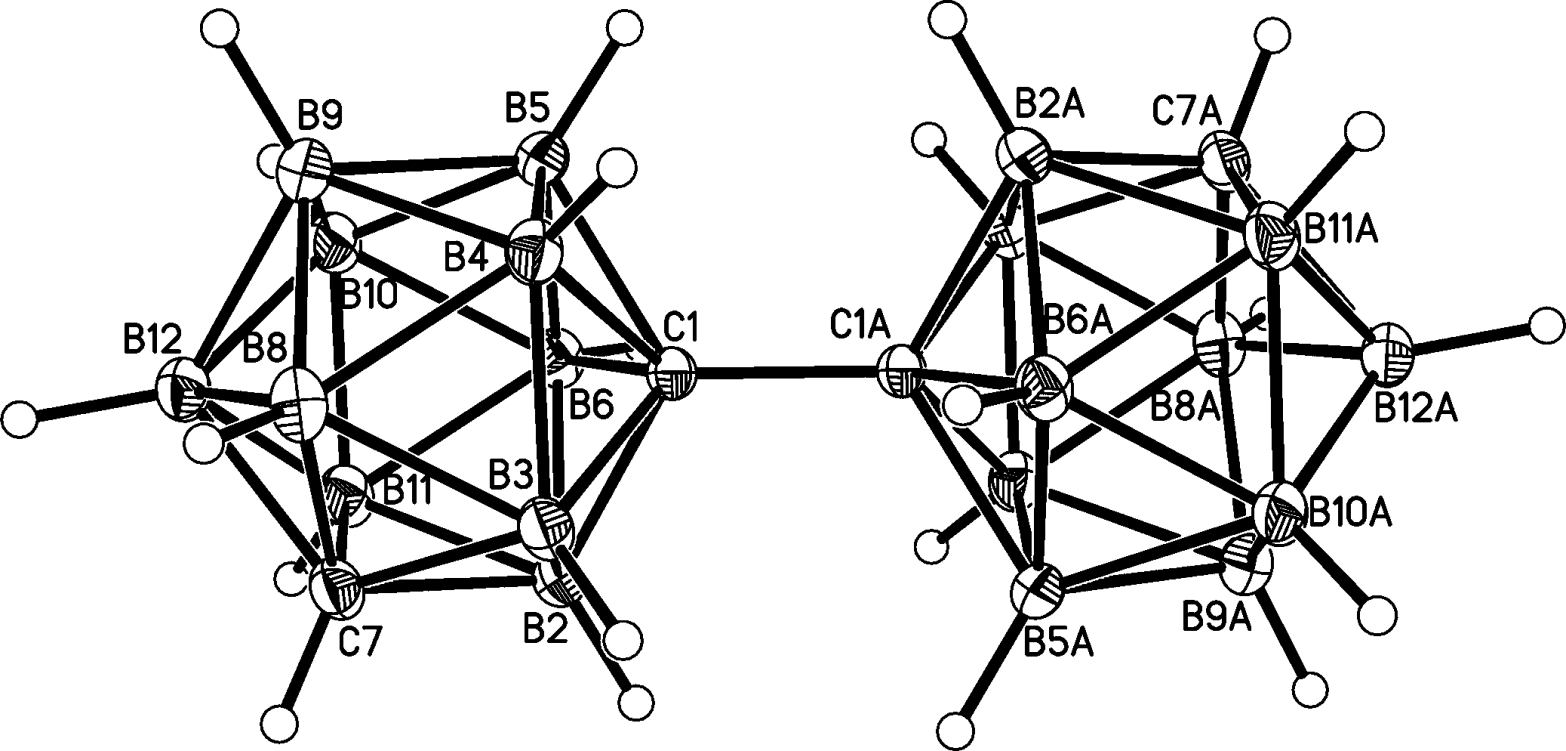
Perspective view of the title compound with displacement ellipsoids drawn at the 50% probability level. The label suffix A refers to the symmetry operation (−*x* + 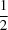, −*y* + 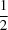, −*z* + 1).

Mol­ecules of (I) are composed of two {1,7-*closo*-C_2_B_10_H_11_} cages (the contents of the asymmetric unit) linked across a crystallographic inversion centre by the C1—C1*A* bond [1.5401 (15) Å; symmetry code: (*A*) ½ − *x*, ½ − *y*, 1 − *z*] (Fig. 1 ▶). The two cages are essentially co-linear, B12⋯C1—C1*A* = 178.72 (7)°, and the facing penta­gons B2/B3/B4/B5/B6 and B2*A*/B3*A*/B4*A*/B5*A*/B6*A* are staggered. The five C1—B distances span the range 1.7107 (12)–1.7385 (12) Å, whilst C7—B connectivities lie between 1.6967 (13) and 1.7180 (13) Å, with, in both cases, the two shortest distances being to the B atoms (B2 and B3) that lie between the C atoms. The B2—B3 connectivity, 1.7947 (13) Å, is the longest B—B link, with all (19) others lying between 1.7709 (13) and 1.7891 (15) Å. In general terms these C—B and B—B distances are fully consistent with the averages recently calculated, 1.705 (14) and 1.772 (11) Å, respectively (McAnaw *et al.*, 2013 ▶), from structural studies of the three carborane isomers 1,2-*closo*-C_2_B_10_H_12_, 1,7-*closo*-C_2_B_10_H_12_ and 1,12-*closo*-C_2_B_10_H_12_ (Davidson *et al.*, 1996 ▶).

## Supra­molecular features   

The only H⋯H contact less than 2.40 Å is H6⋯H6*B* at 2.39 Å [symmetry code: (*B*) −*x* + 1, −*y* + 1, −*z* + 1]. Although CH units and BH units in carboranes are protonic and hydridic, respectively, there is no evidence of di­hydrogen bonding, the shortest such contact being H7⋯H12*C* at 2.61 Å [symmetry code: (*C*) −*x* + 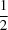, *y* + 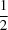, −*z* + 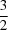].

## Database survey   

A search of the Cambridge Structural Database (Groom & Allen, 2014 ▶) for the 1,7-*closo*-C_2_B_10_ fragment using Conquest (Version 1.16) returned 132 hits of which only two involve the 1,1′-bis­(1,7-dicarba-*closo*-dodeca­borane) unit. In DUWJAH (Stadlbauer *et al.*, 2010 ▶), there are {P(NMe_2_)_2_} groups attached to C7 and C7′ whilst in DUWJEL (Stadlbauer *et al.*, 2010 ▶) these cage atoms are bound to {P(NMe_2_)(OMe)} units. Of the remaining 130 hits there are five cases of the parent mol­ecule 1,7-*closo*-C_2_B_10_H_12_ co-crystallized with other mol­ecules, the first of these to be reported being the hexa­methyl­phospho­r­amide adduct TOKGOP (Davidson *et al.*, 1996 ▶), whilst all others involve either a single 1,7-*closo*-C_2_B_10_ cage with non-H substituents on one or more C or B atoms or multiple cages linked by other than a direct two-centre two-electron bond.

## Synthesis and crystallization   

The compound was prepared by the Cu^I^-mediated coupling of li­thia­ted *meta*-carborane, a method first reported by Yang *et al.* (1992 ▶) for *para*-carborane and later used by Ren & Xie (2008 ▶) for the coupling of *ortho*-carborane. The purity of the product was confirmed by elemental microanalysis and by mass spectrometry and NMR spectroscopy, the last by comparison with data reported by Yang *et al.* (1995 ▶). Colourless plates were afforded by the slow evaporation of a di­chloro­methane solution.

## Refinement   

Crystal data, data collection and structure refinement details are summarized in Table 1 ▶. The complete mol­ecule is generated by a crystallographic centre of symmetry at the mid-point of the C1—C1*A* bond. Initially only the linking atom C1 was identified as a C atom with all other cage atoms described as boron and with H atoms allowed positional refinement. This model (the *Prostructure*) was refined and then analysed by both the *Vertex-to-Centroid Distance* (McAnaw *et al.*, 2013 ▶) and the *Boron–Hydrogen Distance* (McAnaw *et al.*, 2014 ▶) methods. Both methods led to the same unambiguous conclusion regarding the location of the second C atom, C7, and there is no evidence of C/B disorder, a frequent problem in crystallographic studies of carboranes and heterocarboranes. Having identified C7, the refinement was completed with H atoms continuing to be freely refined positionally and with *U*_iso_(H) = 1.2*U*_eq_(C,B).

## Supplementary Material

Crystal structure: contains datablock(s) I. DOI: 10.1107/S1600536814022132/hb7290sup1.cif

Structure factors: contains datablock(s) I. DOI: 10.1107/S1600536814022132/hb7290Isup2.hkl

CCDC reference: 1027936

Additional supporting information:  crystallographic information; 3D view; checkCIF report

## Figures and Tables

**Table 1:** Experimental details

Crystal data	
Chemical formula	C_4_H_22_B_20_
*M*_r_	286.41
Crystal system, space group	Monoclinic, *C*2/*c*
Temperature (K)	100
*a*, *b*, *c* ()	12.1518(13), 6.8308(7), 19.9613(19)
()	93.005(6)
*V* (^3^)	1654.6(3)
*Z*	4
Radiation type	Mo *K*
(mm^1^)	0.05
Crystal size (mm)	0.56 0.38 0.16

Data collection	
Diffractometer	Bruker APEXII CCD
Absorption correction	Multi-scan (*SADABS*; Bruker, 2008 ▶)
*T*_min_, *T*_max_	0.692, 0.747
No. of measured, independent and observed [*I* > 2(*I*)] reflections	19831, 3108, 2261
*R*_int_	0.037
(sin /)_max_ (^1^)	0.767

Refinement	
*R*[*F* ^2^ > 2(*F* ^2^)], *wR*(*F* ^2^), *S*	0.045, 0.130, 1.04
No. of reflections	3108
No. of parameters	142
H-atom treatment	Only H-atom coordinates refined
_max_, _min_ (e ^3^)	0.33, 0.24

Computer programs: *APEX2* and *SAINT* (Bruker, 2009 ▶), *SHELXS97*, *SHELXL97* and *SHELXTL* (Sheldrick, 2008 ▶) and *OLEX2* (Dolomanov *et al.*, 2009 ▶).

## supplementary crystallographic information

### Crystal data

**Table:**

C_4_H_22_B_20_	*F*(000) = 584
*M**_r_* = 286.41	*D*_x_ = 1.150 Mg m^−^^3^
Monoclinic, *C*2/*c*	Mo *K*α radiation, λ = 0.71073 Å
*a* = 12.1518 (13) Å	Cell parameters from 4828 reflections
*b* = 6.8308 (7) Å	θ = 3.4–32.2°
*c* = 19.9613 (19) Å	µ = 0.05 mm^−^^1^
β = 93.005 (6)°	*T* = 100 K
*V* = 1654.6 (3) Å^3^	Plate, colourless
*Z* = 4	0.56 × 0.38 × 0.16 mm

### Data collection

**Table:**

Bruker APEXII CCD diffractometer	2261 reflections with *I* > 2σ(*I*)
Graphite monochromator	*R*_int_ = 0.037
φ and ω scans	θ_max_ = 33.0°, θ_min_ = 3.4°
Absorption correction: multi-scan (*SADABS*; Bruker, 2008)	*h* = −18→18
*T*_min_ = 0.692, *T*_max_ = 0.747	*k* = −10→10
19831 measured reflections	*l* = −30→30
3108 independent reflections	

### Refinement

**Table:**

Refinement on *F*^2^	0 restraints
Least-squares matrix: full	Hydrogen site location: difference Fourier map
*R*[*F*^2^ > 2σ(*F*^2^)] = 0.045	Only H-atom coordinates refined
*wR*(*F*^2^) = 0.130	*w* = 1/[σ^2^(*F*_o_^2^) + (0.0689*P*)^2^ + 0.3243*P*] where *P* = (*F*_o_^2^ + 2*F*_c_^2^)/3
*S* = 1.04	(Δ/σ)_max_ = 0.002
3108 reflections	Δρ_max_ = 0.33 e Å^−^^3^
142 parameters	Δρ_min_ = −0.24 e Å^−^^3^

### Special details

**Table:**

Geometry. All e.s.d.'s (except the e.s.d. in the dihedral angle between two l.s. planes) are estimated using the full covariance matrix. The cell e.s.d.'s are taken into account individually in the estimation of e.s.d.'s in distances, angles and torsion angles; correlations between e.s.d.'s in cell parameters are only used when they are defined by crystal symmetry. An approximate (isotropic) treatment of cell e.s.d.'s is used for estimating e.s.d.'s involving l.s. planes.

### Fractional atomic coordinates and isotropic or equivalent isotropic displacement parameters (Å^2^)

**Table:**

	*x*	*y*	*z*	*U*_iso_*/*U*_eq_	
C1	0.26856 (6)	0.25524 (11)	0.53744 (4)	0.01245 (16)	
B2	0.29131 (8)	0.48296 (14)	0.57171 (5)	0.01579 (19)	
H2	0.2736 (8)	0.6130 (16)	0.5456 (5)	0.019*	
B3	0.17518 (8)	0.33659 (14)	0.59232 (5)	0.01583 (19)	
H3	0.0950 (8)	0.3915 (16)	0.5773 (5)	0.019*	
B4	0.21591 (8)	0.08725 (14)	0.59283 (5)	0.01628 (19)	
H4	0.1605 (8)	−0.0227 (15)	0.5742 (5)	0.020*	
B5	0.35699 (8)	0.07852 (13)	0.57241 (5)	0.01582 (19)	
H5	0.3839 (8)	−0.0388 (15)	0.5406 (5)	0.019*	
B6	0.40335 (7)	0.32253 (14)	0.55914 (5)	0.01591 (19)	
H6	0.4600 (8)	0.3529 (15)	0.5201 (5)	0.019*	
C7	0.25880 (7)	0.44568 (13)	0.65235 (4)	0.01717 (18)	
H7	0.2280 (9)	0.5573 (16)	0.6744 (5)	0.021*	
B8	0.20858 (8)	0.21700 (15)	0.66948 (5)	0.0191 (2)	
H8	0.1434 (9)	0.2019 (15)	0.7044 (6)	0.023*	
B9	0.32124 (9)	0.05629 (15)	0.65735 (5)	0.0194 (2)	
H9	0.3297 (9)	−0.0839 (16)	0.6842 (5)	0.023*	
B10	0.43699 (8)	0.20190 (15)	0.63657 (5)	0.0185 (2)	
H10	0.5214 (9)	0.1591 (17)	0.6502 (5)	0.022*	
B11	0.39611 (8)	0.45205 (14)	0.63588 (5)	0.0178 (2)	
H11	0.4447 (8)	0.5781 (16)	0.6513 (5)	0.021*	
B12	0.34531 (8)	0.28755 (15)	0.69637 (5)	0.0187 (2)	
H12	0.3626 (8)	0.3182 (15)	0.7501 (5)	0.022*	

### Atomic displacement parameters (Å^2^)

**Table:**

	*U*^11^	*U*^22^	*U*^33^	*U*^12^	*U*^13^	*U*^23^
C1	0.0119 (3)	0.0128 (3)	0.0124 (4)	−0.0003 (3)	−0.0010 (3)	−0.0007 (3)
B2	0.0178 (4)	0.0138 (4)	0.0154 (4)	−0.0005 (3)	−0.0024 (3)	−0.0020 (3)
B3	0.0145 (4)	0.0191 (4)	0.0138 (4)	0.0009 (3)	−0.0001 (3)	−0.0030 (3)
B4	0.0177 (4)	0.0170 (4)	0.0140 (4)	−0.0025 (3)	−0.0001 (3)	0.0010 (3)
B5	0.0165 (4)	0.0154 (4)	0.0151 (4)	0.0026 (3)	−0.0031 (3)	−0.0006 (3)
B6	0.0124 (4)	0.0190 (4)	0.0160 (4)	−0.0012 (3)	−0.0016 (3)	−0.0020 (3)
C7	0.0167 (4)	0.0201 (4)	0.0144 (4)	0.0023 (3)	−0.0020 (3)	−0.0044 (3)
B8	0.0207 (5)	0.0229 (5)	0.0137 (4)	−0.0019 (4)	0.0002 (4)	−0.0009 (3)
B9	0.0250 (5)	0.0186 (4)	0.0142 (4)	0.0019 (3)	−0.0020 (4)	0.0008 (3)
B10	0.0165 (4)	0.0219 (5)	0.0167 (5)	0.0037 (3)	−0.0042 (3)	−0.0034 (3)
B11	0.0156 (4)	0.0203 (4)	0.0169 (5)	−0.0009 (3)	−0.0033 (3)	−0.0034 (3)
B12	0.0197 (4)	0.0212 (4)	0.0147 (4)	0.0030 (3)	−0.0028 (3)	−0.0019 (3)

### Geometric parameters (Å, º)

**Table:**

C1—C1^i^	1.5401 (15)	B5—B9	1.7786 (14)
C1—B2	1.7158 (12)	B5—B10	1.7790 (13)
C1—B3	1.7107 (12)	B6—H6	1.086 (11)
C1—B4	1.7385 (12)	B6—B10	1.7802 (14)
C1—B5	1.7380 (12)	B6—B11	1.7751 (14)
C1—B6	1.7342 (12)	C7—H7	0.966 (11)
B2—H2	1.046 (11)	C7—B8	1.7179 (13)
B2—B3	1.7947 (13)	C7—B11	1.7180 (13)
B2—B6	1.7758 (13)	C7—B12	1.7167 (13)
B2—C7	1.6967 (13)	B8—H8	1.087 (11)
B2—B11	1.7709 (13)	B8—B9	1.7812 (14)
B3—H3	1.072 (10)	B8—B12	1.7854 (14)
B3—B4	1.7735 (13)	B9—H9	1.099 (11)
B3—C7	1.7017 (13)	B9—B10	1.7891 (15)
B3—B8	1.7717 (14)	B9—B12	1.7787 (14)
B4—H4	1.063 (10)	B10—H10	1.087 (10)
B4—B5	1.7839 (13)	B10—B11	1.7794 (14)
B4—B8	1.7745 (14)	B10—B12	1.7742 (14)
B4—B9	1.7802 (14)	B11—H11	1.079 (11)
B5—H5	1.084 (10)	B11—B12	1.7832 (14)
B5—B6	1.7836 (13)	B12—H12	1.103 (11)
			
C1^i^—C1—B2	117.51 (8)	B2—C7—B3	63.76 (5)
C1^i^—C1—B3	117.79 (8)	B2—C7—H7	115.4 (6)
C1^i^—C1—B4	119.19 (8)	B2—C7—B8	115.35 (7)
C1^i^—C1—B5	120.07 (8)	B2—C7—B11	62.48 (5)
C1^i^—C1—B6	118.65 (8)	B2—C7—B12	114.41 (7)
B2—C1—B4	113.69 (6)	B3—C7—H7	115.8 (6)
B2—C1—B5	112.72 (6)	B3—C7—B8	62.41 (6)
B2—C1—B6	61.95 (5)	B3—C7—B11	115.24 (6)
B3—C1—B2	63.17 (5)	B3—C7—B12	114.33 (7)
B3—C1—B4	61.88 (5)	B8—C7—H7	118.5 (6)
B3—C1—B5	112.66 (6)	B8—C7—B11	114.82 (7)
B3—C1—B6	113.79 (6)	B11—C7—H7	118.1 (6)
B5—C1—B4	61.74 (5)	B12—C7—H7	120.3 (7)
B6—C1—B4	112.98 (6)	B12—C7—B8	62.64 (6)
B6—C1—B5	61.82 (5)	B12—C7—B11	62.55 (6)
C1—B2—H2	123.1 (6)	B3—B8—B4	60.01 (5)
C1—B2—B3	58.28 (5)	B3—B8—H8	117.4 (6)
C1—B2—B6	59.53 (5)	B3—B8—B9	107.98 (7)
C1—B2—B11	105.96 (6)	B3—B8—B12	107.70 (7)
B3—B2—H2	116.3 (5)	B4—B8—H8	125.3 (6)
B6—B2—H2	126.3 (5)	B4—B8—B9	60.09 (6)
B6—B2—B3	107.85 (7)	B4—B8—B12	107.82 (7)
C7—B2—C1	101.62 (6)	C7—B8—B3	58.35 (5)
C7—B2—H2	123.3 (6)	C7—B8—B4	104.36 (7)
C7—B2—B3	58.26 (5)	C7—B8—H8	119.5 (6)
C7—B2—B6	105.06 (7)	C7—B8—B9	104.46 (7)
C7—B2—B11	59.35 (5)	C7—B8—B12	58.65 (5)
B11—B2—H2	125.6 (6)	B9—B8—H8	128.3 (6)
B11—B2—B3	108.18 (7)	B9—B8—B12	59.83 (6)
B11—B2—B6	60.06 (5)	B12—B8—H8	122.0 (6)
C1—B3—B2	58.55 (5)	B4—B9—B8	59.77 (5)
C1—B3—H3	124.0 (5)	B4—B9—H9	120.1 (6)
C1—B3—B4	59.83 (5)	B4—B9—B10	108.09 (7)
C1—B3—B8	106.11 (7)	B5—B9—B4	60.17 (5)
B2—B3—H3	117.1 (6)	B5—B9—B8	107.75 (7)
B4—B3—B2	108.29 (6)	B5—B9—H9	121.1 (6)
B4—B3—H3	126.0 (6)	B5—B9—B10	59.82 (6)
C7—B3—C1	101.63 (6)	B5—B9—B12	107.39 (7)
C7—B3—B2	57.99 (5)	B8—B9—H9	121.6 (6)
C7—B3—H3	122.9 (6)	B8—B9—B10	107.95 (7)
C7—B3—B4	105.09 (7)	B10—B9—H9	122.8 (6)
C7—B3—B8	59.24 (5)	B12—B9—B4	107.87 (7)
B8—B3—B2	108.01 (7)	B12—B9—B8	60.20 (6)
B8—B3—H3	124.6 (6)	B12—B9—H9	123.5 (6)
B8—B3—B4	60.07 (5)	B12—B9—B10	59.64 (6)
C1—B4—B3	58.29 (5)	B5—B10—B6	60.15 (5)
C1—B4—H4	119.4 (6)	B5—B10—B9	59.80 (5)
C1—B4—B5	59.11 (5)	B5—B10—H10	121.7 (6)
C1—B4—B8	104.80 (6)	B5—B10—B11	107.91 (7)
C1—B4—B9	105.30 (6)	B6—B10—B9	108.12 (7)
B3—B4—H4	120.4 (6)	B6—B10—H10	120.9 (6)
B3—B4—B5	107.58 (6)	B9—B10—H10	122.4 (6)
B3—B4—B8	59.91 (5)	B11—B10—B6	59.83 (5)
B3—B4—B9	107.94 (7)	B11—B10—B9	108.24 (7)
B5—B4—H4	119.6 (6)	B11—B10—H10	121.3 (6)
B8—B4—H4	126.6 (6)	B12—B10—B5	107.57 (7)
B8—B4—B5	107.81 (7)	B12—B10—B6	107.92 (7)
B8—B4—B9	60.14 (6)	B12—B10—B9	59.89 (6)
B9—B4—H4	126.0 (6)	B12—B10—H10	122.5 (6)
B9—B4—B5	59.87 (5)	B12—B10—B11	60.24 (6)
C1—B5—B4	59.14 (5)	B2—B11—B6	60.10 (5)
C1—B5—H5	118.5 (5)	B2—B11—B10	108.06 (7)
C1—B5—B6	58.98 (5)	B2—B11—H11	118.2 (6)
C1—B5—B9	105.39 (6)	B2—B11—B12	107.67 (7)
C1—B5—B10	105.26 (6)	B6—B11—B10	60.11 (5)
B4—B5—H5	118.8 (5)	B6—B11—H11	126.3 (6)
B6—B5—B4	108.51 (6)	B6—B11—B12	107.75 (7)
B6—B5—H5	119.8 (5)	C7—B11—B2	58.17 (5)
B9—B5—B4	59.96 (5)	C7—B11—B6	104.18 (6)
B9—B5—H5	126.3 (5)	C7—B11—B10	104.34 (7)
B9—B5—B6	108.43 (7)	C7—B11—H11	119.2 (6)
B9—B5—B10	60.38 (6)	C7—B11—B12	58.69 (5)
B10—B5—B4	108.37 (7)	B10—B11—H11	128.0 (6)
B10—B5—H5	127.0 (5)	B10—B11—B12	59.73 (6)
B10—B5—B6	59.96 (5)	B12—B11—H11	120.9 (6)
C1—B6—B2	58.52 (5)	C7—B12—B8	58.71 (5)
C1—B6—B5	59.20 (5)	C7—B12—B9	104.62 (7)
C1—B6—H6	119.8 (5)	C7—B12—B10	104.62 (7)
C1—B6—B10	105.37 (7)	C7—B12—B11	58.76 (5)
C1—B6—B11	105.00 (6)	C7—B12—H12	117.3 (6)
B2—B6—B5	107.77 (6)	B8—B12—H12	118.1 (5)
B2—B6—H6	120.2 (6)	B9—B12—B8	59.97 (6)
B2—B6—B10	107.81 (7)	B9—B12—B11	108.54 (7)
B5—B6—H6	120.1 (6)	B9—B12—H12	127.8 (6)
B10—B6—B5	59.89 (5)	B10—B12—B8	108.42 (7)
B10—B6—H6	125.9 (5)	B10—B12—B9	60.47 (6)
B11—B6—B2	59.83 (5)	B10—B12—B11	60.02 (6)
B11—B6—B5	107.90 (7)	B10—B12—H12	128.5 (5)
B11—B6—H6	125.9 (6)	B11—B12—B8	108.44 (7)
B11—B6—B10	60.07 (5)	B11—B12—H12	119.0 (6)
			
C1^i^—C1—B2—B3	109.00 (9)	B4—B9—B12—C7	−2.16 (9)
C1^i^—C1—B2—B6	−109.58 (9)	B4—B9—B12—B8	37.42 (7)
C1^i^—C1—B2—C7	149.66 (8)	B4—B9—B12—B10	−100.91 (7)
C1^i^—C1—B2—B11	−149.18 (8)	B4—B9—B12—B11	−63.62 (8)
C1^i^—C1—B3—B2	−108.57 (9)	B5—C1—B2—B3	−104.85 (7)
C1^i^—C1—B3—B4	110.08 (9)	B5—C1—B2—B6	36.57 (6)
C1^i^—C1—B3—C7	−149.09 (8)	B5—C1—B2—C7	−64.19 (8)
C1^i^—C1—B3—B8	149.86 (8)	B5—C1—B2—B11	−3.04 (8)
C1^i^—C1—B4—B3	−107.87 (9)	B5—C1—B3—B2	104.94 (7)
C1^i^—C1—B4—B5	110.57 (10)	B5—C1—B3—B4	−36.40 (6)
C1^i^—C1—B4—B8	−147.27 (9)	B5—C1—B3—C7	64.43 (8)
C1^i^—C1—B4—B9	150.21 (8)	B5—C1—B3—B8	3.37 (8)
C1^i^—C1—B5—B4	−109.18 (9)	B5—C1—B4—B3	141.56 (7)
C1^i^—C1—B5—B6	108.56 (10)	B5—C1—B4—B8	102.16 (7)
C1^i^—C1—B5—B9	−148.89 (9)	B5—C1—B4—B9	39.65 (6)
C1^i^—C1—B5—B10	148.25 (8)	B5—C1—B6—B2	−141.43 (7)
C1^i^—C1—B6—B2	107.77 (9)	B5—C1—B6—B10	−39.68 (6)
C1^i^—C1—B6—B5	−110.79 (10)	B5—C1—B6—B11	−102.16 (7)
C1^i^—C1—B6—B10	−150.48 (8)	B5—B4—B8—B3	100.40 (7)
C1^i^—C1—B6—B11	147.05 (9)	B5—B4—B8—C7	61.04 (8)
C1—B2—B3—B4	34.67 (6)	B5—B4—B8—B9	−37.60 (7)
C1—B2—B3—C7	131.37 (7)	B5—B4—B8—B12	−0.16 (9)
C1—B2—B3—B8	98.24 (7)	B5—B4—B9—B8	137.81 (7)
C1—B2—B6—B5	−34.22 (6)	B5—B4—B9—B10	37.16 (6)
C1—B2—B6—B10	−97.46 (7)	B5—B4—B9—B12	100.20 (8)
C1—B2—B6—B11	−134.99 (7)	B5—B6—B10—B9	−37.05 (6)
C1—B2—C7—B3	−40.67 (6)	B5—B6—B10—B11	−138.05 (7)
C1—B2—C7—B8	−4.28 (8)	B5—B6—B10—B12	−100.38 (7)
C1—B2—C7—B11	101.80 (7)	B5—B6—B11—B2	−100.57 (7)
C1—B2—C7—B12	65.63 (8)	B5—B6—B11—C7	−61.17 (8)
C1—B2—B11—B6	39.35 (6)	B5—B6—B11—B10	37.42 (6)
C1—B2—B11—C7	−94.25 (7)	B5—B6—B11—B12	0.02 (9)
C1—B2—B11—B10	1.73 (8)	B5—B9—B10—B6	37.21 (6)
C1—B2—B11—B12	−61.36 (8)	B5—B9—B10—B11	100.53 (7)
C1—B3—B4—B5	34.03 (6)	B5—B9—B10—B12	137.84 (7)
C1—B3—B4—B8	134.83 (7)	B5—B9—B12—C7	61.30 (8)
C1—B3—B4—B9	97.24 (7)	B5—B9—B12—B8	100.89 (7)
C1—B3—C7—B2	40.82 (6)	B5—B9—B12—B10	−37.44 (6)
C1—B3—C7—B8	−101.96 (7)	B5—B9—B12—B11	−0.15 (9)
C1—B3—C7—B11	4.14 (9)	B5—B10—B11—B2	0.07 (9)
C1—B3—C7—B12	−65.61 (8)	B5—B10—B11—B6	−37.54 (6)
C1—B3—B8—B4	−39.66 (6)	B5—B10—B11—C7	60.79 (8)
C1—B3—B8—C7	94.14 (7)	B5—B10—B11—B12	100.41 (7)
C1—B3—B8—B9	−2.08 (9)	B5—B10—B12—C7	−61.27 (8)
C1—B3—B8—B12	61.11 (8)	B5—B10—B12—B8	0.13 (9)
C1—B4—B5—B6	33.59 (6)	B5—B10—B12—B9	37.48 (6)
C1—B4—B5—B9	134.64 (7)	B5—B10—B12—B11	−100.98 (7)
C1—B4—B5—B10	97.16 (7)	B6—C1—B2—B3	−141.42 (7)
C1—B4—B8—B3	38.61 (6)	B6—C1—B2—C7	−100.76 (7)
C1—B4—B8—C7	−0.75 (8)	B6—C1—B2—B11	−39.60 (6)
C1—B4—B8—B9	−99.38 (7)	B6—C1—B3—B2	36.98 (7)
C1—B4—B8—B12	−61.94 (8)	B6—C1—B3—B4	−104.36 (7)
C1—B4—B9—B5	−39.28 (6)	B6—C1—B3—C7	−3.54 (9)
C1—B4—B9—B8	98.53 (7)	B6—C1—B3—B8	−64.59 (8)
C1—B4—B9—B10	−2.12 (8)	B6—C1—B4—B3	105.68 (7)
C1—B4—B9—B12	60.92 (8)	B6—C1—B4—B5	−35.88 (6)
C1—B5—B6—B2	33.94 (6)	B6—C1—B4—B8	66.28 (8)
C1—B5—B6—B10	134.63 (7)	B6—C1—B4—B9	3.77 (9)
C1—B5—B6—B11	97.13 (7)	B6—C1—B5—B4	142.25 (7)
C1—B5—B9—B4	39.31 (6)	B6—C1—B5—B9	102.54 (7)
C1—B5—B9—B8	1.78 (9)	B6—C1—B5—B10	39.69 (6)
C1—B5—B9—B10	−99.06 (7)	B6—B2—B3—C1	−34.38 (6)
C1—B5—B9—B12	−61.70 (8)	B6—B2—B3—B4	0.29 (9)
C1—B5—B10—B6	−39.22 (6)	B6—B2—B3—C7	96.99 (7)
C1—B5—B10—B9	99.28 (7)	B6—B2—B3—B8	63.86 (8)
C1—B5—B10—B11	−1.82 (9)	B6—B2—C7—B3	−101.93 (7)
C1—B5—B10—B12	61.77 (8)	B6—B2—C7—B8	−65.55 (8)
C1—B6—B10—B5	39.35 (6)	B6—B2—C7—B11	40.53 (6)
C1—B6—B10—B9	2.29 (9)	B6—B2—C7—B12	4.36 (9)
C1—B6—B10—B11	−98.71 (7)	B6—B2—B11—C7	−133.60 (7)
C1—B6—B10—B12	−61.03 (8)	B6—B2—B11—B10	−37.61 (6)
C1—B6—B11—B2	−38.64 (6)	B6—B2—B11—B12	−100.71 (7)
C1—B6—B11—C7	0.75 (8)	B6—B5—B9—B4	101.17 (7)
C1—B6—B11—B10	99.35 (7)	B6—B5—B9—B8	63.64 (8)
C1—B6—B11—B12	61.94 (8)	B6—B5—B9—B10	−37.20 (6)
B2—C1—B3—B4	−141.34 (7)	B6—B5—B9—B12	0.16 (9)
B2—C1—B3—C7	−40.52 (6)	B6—B5—B10—B9	138.50 (7)
B2—C1—B3—B8	−101.57 (7)	B6—B5—B10—B11	37.39 (6)
B2—C1—B4—B3	37.50 (6)	B6—B5—B10—B12	100.98 (7)
B2—C1—B4—B5	−104.06 (7)	B6—B10—B11—B2	37.61 (6)
B2—C1—B4—B8	−1.90 (9)	B6—B10—B11—C7	98.32 (7)
B2—C1—B4—B9	−64.42 (8)	B6—B10—B11—B12	137.94 (7)
B2—C1—B5—B4	105.64 (7)	B6—B10—B12—C7	2.23 (9)
B2—C1—B5—B6	−36.62 (6)	B6—B10—B12—B8	63.62 (9)
B2—C1—B5—B9	65.92 (8)	B6—B10—B12—B9	100.97 (7)
B2—C1—B5—B10	3.07 (9)	B6—B10—B12—B11	−37.49 (6)
B2—C1—B6—B5	141.43 (7)	B6—B11—B12—C7	−96.11 (7)
B2—C1—B6—B10	101.75 (7)	B6—B11—B12—B8	−63.51 (8)
B2—C1—B6—B11	39.27 (6)	B6—B11—B12—B9	0.08 (9)
B2—B3—B4—C1	−34.14 (6)	B6—B11—B12—B10	37.57 (6)
B2—B3—B4—B5	−0.11 (9)	C7—B2—B3—C1	−131.37 (7)
B2—B3—B4—B8	100.68 (7)	C7—B2—B3—B4	−96.70 (7)
B2—B3—B4—B9	63.09 (8)	C7—B2—B3—B8	−33.13 (6)
B2—B3—C7—B8	−142.78 (7)	C7—B2—B6—C1	94.81 (7)
B2—B3—C7—B11	−36.68 (7)	C7—B2—B6—B5	60.59 (8)
B2—B3—C7—B12	−106.43 (7)	C7—B2—B6—B10	−2.65 (8)
B2—B3—B8—B4	−101.16 (7)	C7—B2—B6—B11	−40.18 (6)
B2—B3—B8—C7	32.64 (6)	C7—B2—B11—B6	133.60 (7)
B2—B3—B8—B9	−63.59 (8)	C7—B2—B11—B10	95.98 (7)
B2—B3—B8—B12	−0.40 (9)	C7—B2—B11—B12	32.89 (6)
B2—B6—B10—B5	100.62 (7)	C7—B3—B4—C1	−94.86 (7)
B2—B6—B10—B9	63.57 (8)	C7—B3—B4—B5	−60.82 (8)
B2—B6—B10—B11	−37.43 (6)	C7—B3—B4—B8	39.97 (6)
B2—B6—B10—B12	0.24 (9)	C7—B3—B4—B9	2.38 (8)
B2—B6—B11—C7	39.39 (6)	C7—B3—B8—B4	−133.80 (7)
B2—B6—B11—B10	137.99 (7)	C7—B3—B8—B9	−96.23 (7)
B2—B6—B11—B12	100.58 (7)	C7—B3—B8—B12	−33.04 (6)
B2—C7—B8—B3	−36.90 (7)	C7—B8—B9—B4	−98.46 (7)
B2—C7—B8—B4	3.29 (9)	C7—B8—B9—B5	−60.75 (8)
B2—C7—B8—B9	65.54 (8)	C7—B8—B9—B10	2.43 (9)
B2—C7—B8—B12	105.65 (7)	C7—B8—B9—B12	39.52 (6)
B2—C7—B11—B6	−40.36 (6)	C7—B8—B12—B9	−133.82 (7)
B2—C7—B11—B10	−102.59 (7)	C7—B8—B12—B10	−96.25 (7)
B2—C7—B11—B12	−142.73 (7)	C7—B8—B12—B11	−32.62 (6)
B2—C7—B12—B8	−107.13 (7)	C7—B11—B12—B8	32.60 (6)
B2—C7—B12—B9	−66.93 (9)	C7—B11—B12—B9	96.20 (7)
B2—C7—B12—B10	−4.21 (9)	C7—B11—B12—B10	133.68 (7)
B2—C7—B12—B11	36.14 (7)	B8—B3—B4—C1	−134.83 (7)
B2—B11—B12—C7	−32.68 (6)	B8—B3—B4—B5	−100.79 (7)
B2—B11—B12—B8	−0.09 (9)	B8—B3—B4—B9	−37.59 (6)
B2—B11—B12—B9	63.51 (8)	B8—B3—C7—B2	142.78 (7)
B2—B11—B12—B10	101.00 (7)	B8—B3—C7—B11	106.10 (8)
B3—C1—B2—B6	141.42 (7)	B8—B3—C7—B12	36.35 (7)
B3—C1—B2—C7	40.66 (6)	B8—B4—B5—C1	−96.92 (7)
B3—C1—B2—B11	101.82 (7)	B8—B4—B5—B6	−63.33 (8)
B3—C1—B4—B5	−141.56 (7)	B8—B4—B5—B9	37.72 (6)
B3—C1—B4—B8	−39.40 (6)	B8—B4—B5—B10	0.24 (9)
B3—C1—B4—B9	−101.91 (7)	B8—B4—B9—B5	−137.81 (7)
B3—C1—B5—B4	36.45 (6)	B8—B4—B9—B10	−100.65 (7)
B3—C1—B5—B6	−105.80 (7)	B8—B4—B9—B12	−37.61 (7)
B3—C1—B5—B9	−3.26 (8)	B8—C7—B11—B2	106.91 (7)
B3—C1—B5—B10	−66.11 (8)	B8—C7—B11—B6	66.55 (9)
B3—C1—B6—B2	−37.46 (7)	B8—C7—B11—B10	4.32 (9)
B3—C1—B6—B5	103.98 (7)	B8—C7—B11—B12	−35.82 (7)
B3—C1—B6—B10	64.29 (8)	B8—C7—B12—B9	40.20 (6)
B3—C1—B6—B11	1.82 (9)	B8—C7—B12—B10	102.92 (7)
B3—B2—B6—C1	33.87 (6)	B8—C7—B12—B11	143.27 (7)
B3—B2—B6—B5	−0.35 (9)	B8—B9—B10—B5	−100.51 (7)
B3—B2—B6—B10	−63.59 (8)	B8—B9—B10—B6	−63.30 (8)
B3—B2—B6—B11	−101.12 (7)	B8—B9—B10—B11	0.02 (9)
B3—B2—C7—B8	36.39 (7)	B8—B9—B10—B12	37.33 (6)
B3—B2—C7—B11	142.47 (7)	B8—B9—B12—C7	−39.58 (6)
B3—B2—C7—B12	106.30 (8)	B8—B9—B12—B10	−138.33 (7)
B3—B2—B11—B6	100.55 (7)	B8—B9—B12—B11	−101.04 (7)
B3—B2—B11—C7	−33.05 (6)	B9—B4—B5—C1	−134.64 (7)
B3—B2—B11—B10	62.94 (8)	B9—B4—B5—B6	−101.05 (7)
B3—B2—B11—B12	−0.16 (9)	B9—B4—B5—B10	−37.48 (6)
B3—B4—B5—C1	−33.70 (6)	B9—B4—B8—B3	138.00 (7)
B3—B4—B5—B6	−0.11 (9)	B9—B4—B8—C7	98.63 (7)
B3—B4—B5—B9	100.94 (7)	B9—B4—B8—B12	37.44 (7)
B3—B4—B5—B10	63.46 (8)	B9—B5—B6—C1	−97.24 (7)
B3—B4—B8—C7	−39.36 (6)	B9—B5—B6—B2	−63.30 (8)
B3—B4—B8—B9	−138.00 (7)	B9—B5—B6—B10	37.39 (6)
B3—B4—B8—B12	−100.56 (7)	B9—B5—B6—B11	−0.11 (8)
B3—B4—B9—B5	−100.32 (7)	B9—B5—B10—B6	−138.50 (7)
B3—B4—B9—B8	37.49 (6)	B9—B5—B10—B11	−101.10 (7)
B3—B4—B9—B10	−63.16 (8)	B9—B5—B10—B12	−37.52 (7)
B3—B4—B9—B12	−0.12 (9)	B9—B8—B12—C7	133.82 (7)
B3—C7—B8—B4	40.19 (6)	B9—B8—B12—B10	37.57 (7)
B3—C7—B8—B9	102.44 (7)	B9—B8—B12—B11	101.21 (7)
B3—C7—B8—B12	142.55 (7)	B9—B10—B11—B2	−63.17 (8)
B3—C7—B11—B2	37.16 (7)	B9—B10—B11—B6	−100.78 (7)
B3—C7—B11—B6	−3.20 (9)	B9—B10—B11—C7	−2.46 (8)
B3—C7—B11—B10	−65.42 (9)	B9—B10—B11—B12	37.16 (6)
B3—C7—B11—B12	−105.57 (8)	B9—B10—B12—C7	−98.74 (7)
B3—C7—B12—B8	−36.26 (7)	B9—B10—B12—B8	−37.35 (7)
B3—C7—B12—B9	3.94 (9)	B9—B10—B12—B11	−138.46 (7)
B3—C7—B12—B10	66.66 (9)	B10—B5—B6—C1	−134.63 (7)
B3—C7—B12—B11	107.01 (7)	B10—B5—B6—B2	−100.69 (7)
B3—B8—B9—B4	−37.54 (6)	B10—B5—B6—B11	−37.50 (6)
B3—B8—B9—B5	0.17 (9)	B10—B5—B9—B4	138.38 (7)
B3—B8—B9—B10	63.35 (8)	B10—B5—B9—B8	100.84 (7)
B3—B8—B9—B12	100.44 (7)	B10—B5—B9—B12	37.36 (6)
B3—B8—B12—C7	32.92 (6)	B10—B6—B11—B2	−137.99 (7)
B3—B8—B12—B9	−100.91 (7)	B10—B6—B11—C7	−98.59 (7)
B3—B8—B12—B10	−63.34 (9)	B10—B6—B11—B12	−37.41 (6)
B3—B8—B12—B11	0.30 (9)	B10—B9—B12—C7	98.75 (7)
B4—C1—B2—B3	−36.99 (6)	B10—B9—B12—B8	138.33 (7)
B4—C1—B2—B6	104.43 (7)	B10—B9—B12—B11	37.29 (6)
B4—C1—B2—C7	3.67 (8)	B10—B11—B12—C7	−133.68 (7)
B4—C1—B2—B11	64.83 (8)	B10—B11—B12—B8	−101.09 (7)
B4—C1—B3—B2	141.34 (7)	B10—B11—B12—B9	−37.49 (6)
B4—C1—B3—C7	100.83 (7)	B11—B2—B3—C1	−97.89 (7)
B4—C1—B3—B8	39.78 (6)	B11—B2—B3—B4	−63.22 (8)
B4—C1—B5—B6	−142.25 (7)	B11—B2—B3—C7	33.48 (6)
B4—C1—B5—B9	−39.71 (6)	B11—B2—B3—B8	0.35 (9)
B4—C1—B5—B10	−102.57 (7)	B11—B2—B6—C1	134.99 (7)
B4—C1—B6—B2	−105.58 (7)	B11—B2—B6—B5	100.77 (7)
B4—C1—B6—B5	35.85 (6)	B11—B2—B6—B10	37.53 (6)
B4—C1—B6—B10	−3.83 (9)	B11—B2—C7—B3	−142.47 (7)
B4—C1—B6—B11	−66.31 (8)	B11—B2—C7—B8	−106.08 (7)
B4—B3—C7—B2	102.40 (7)	B11—B2—C7—B12	−36.17 (7)
B4—B3—C7—B8	−40.38 (6)	B11—B6—B10—B5	138.05 (7)
B4—B3—C7—B11	65.72 (8)	B11—B6—B10—B9	101.00 (7)
B4—B3—C7—B12	−4.03 (9)	B11—B6—B10—B12	37.67 (6)
B4—B3—B8—C7	133.80 (7)	B11—C7—B8—B3	−106.76 (7)
B4—B3—B8—B9	37.58 (6)	B11—C7—B8—B4	−66.57 (9)
B4—B3—B8—B12	100.77 (7)	B11—C7—B8—B9	−4.32 (9)
B4—B5—B6—C1	−33.65 (6)	B11—C7—B8—B12	35.79 (7)
B4—B5—B6—B2	0.28 (9)	B11—C7—B12—B8	−143.27 (7)
B4—B5—B6—B10	100.97 (7)	B11—C7—B12—B9	−103.06 (7)
B4—B5—B6—B11	63.47 (8)	B11—C7—B12—B10	−40.35 (6)
B4—B5—B9—B8	−37.54 (6)	B11—B10—B12—C7	39.72 (6)
B4—B5—B9—B10	−138.38 (7)	B11—B10—B12—B8	101.11 (7)
B4—B5—B9—B12	−101.01 (7)	B11—B10—B12—B9	138.46 (7)
B4—B5—B10—B6	−101.21 (7)	B12—C7—B8—B3	−142.55 (7)
B4—B5—B10—B9	37.29 (6)	B12—C7—B8—B4	−102.36 (7)
B4—B5—B10—B11	−63.81 (8)	B12—C7—B8—B9	−40.10 (6)
B4—B5—B10—B12	−0.22 (9)	B12—C7—B11—B2	142.73 (7)
B4—B8—B9—B5	37.71 (6)	B12—C7—B11—B6	102.37 (7)
B4—B8—B9—B10	100.90 (7)	B12—C7—B11—B10	40.14 (6)
B4—B8—B9—B12	137.98 (7)	B12—B8—B9—B4	−137.98 (7)
B4—B8—B12—C7	96.27 (7)	B12—B8—B9—B5	−100.27 (8)
B4—B8—B12—B9	−37.55 (6)	B12—B8—B9—B10	−37.08 (6)
B4—B8—B12—B10	0.02 (9)	B12—B9—B10—B5	−137.84 (7)
B4—B8—B12—B11	63.66 (9)	B12—B9—B10—B6	−100.64 (7)
B4—B9—B10—B5	−37.31 (6)	B12—B9—B10—B11	−37.31 (6)
B4—B9—B10—B6	−0.11 (9)	B12—B10—B11—B2	−100.33 (7)
B4—B9—B10—B11	63.22 (8)	B12—B10—B11—B6	−137.94 (7)
B4—B9—B10—B12	100.53 (7)	B12—B10—B11—C7	−39.62 (6)

Symmetry code: (i) −*x*+1/2, −*y*+1/2, −*z*+1.
